# Immediate versus delayed loading of strategic mini dental implants for the stabilization of partial removable dental prostheses: a patient cluster randomized, parallel-group 3-year trial

**DOI:** 10.1186/s12903-016-0259-z

**Published:** 2016-07-30

**Authors:** Torsten Mundt, Ahmad Al Jaghsi, Bernd Schwahn, Janina Hilgert, Christian Lucas, Reiner Biffar, Christian Schwahn, Friedhelm Heinemann

**Affiliations:** 1 Department of Prosthodontics, Greifswald University Hospital, Gerodontology and Dental Materials, Greifswald, Germany; 2 Private Practice, Greifswald, Germany; 3 Private Practice, Drensteinfurt, Germany; 4 Department of Oral and Maxillofacial Surgery/Plastic Surgery, Greifswald University Hospital, Greifswald, Germany; 5 Department of Prosthodontics, Gerodontology and Dental Materials, Private Practice, Morsbach-Lichtenberg, Germany and Greifswald University Hospital, Greifswald, Germany

**Keywords:** Mini dental implant, Strategic implant, Partial removable dental prosthesis, Supplementary abutment, Implant therapy

## Abstract

**Background:**

Acceptable short-term survival rates (>90 %) of mini-implants (diameter < 3.0 mm) are only documented for mandibular overdentures. Sound data for mini-implants as strategic abutments for a better retention of partial removable dental prosthesis (PRDP) are not available.

**Methods/design:**

The purpose of this study is to test the hypothesis that immediately loaded mini-implants show more bone loss and less success than strategic mini-implants with delayed loading. In this four-center (one university hospital, three dental practices in Germany), parallel-group, controlled clinical trial, which is cluster randomized on patient level, a total of 80 partially edentulous patients with unfavourable number and distribution of remaining abutment teeth in at least one jaw will receive supplementary min-implants to stabilize their PRDP. The mini-implant are either immediately loaded after implant placement (test group) or delayed after four months (control group). Follow-up of the patients will be performed for 36 months. The primary outcome is the radiographic bone level changes at implants. The secondary outcome is the implant success as a composite variable. Tertiary outcomes include clinical, subjective (quality of life, satisfaction, chewing ability) and dental or technical complications.

**Discussion:**

Strategic implants under an existing PRDP are only documented for standard-diameter implants. Mini-implants could be a minimal invasive and low cost solution for this treatment modality.

**Trial registration:**

The trial is registered at Deutsches Register Klinischer Studien (German register of clinical trials) under DRKS-ID: DRKS00007589 (www.germanctr.de) on January 13^th^, 2015.

**Electronic supplementary material:**

The online version of this article (doi:10.1186/s12903-016-0259-z) contains supplementary material, which is available to authorized users.

## Background

In the USA and many European countries there is a decline in edentulism in all age groups. However, the number of partially edentulous patients will increase with a shift in older age groups because the populations of industrial nations are growing older [1, 2]. In Germany, most individuals with reduced dentition demand and receive partial removable dental prostheses (PRDP) that are either retained by double crowns, clasps, ball- or other attachments [3]. The longevity of the remaining teeth and the PRDP depends on the number and localization of the abutments as shown in studies that analysed double crown retained prostheses [4]. According to epidemiological studies, the lower the number of remaining teeth the higher is the incidence of further tooth loss [5]. From the clinical point of view, symmetrical support by the abutment teeth is suggested regardless of the attachment system used [6]. Supplementary implants in strategic positions can ensure a change from a critical prosthetic support (unilateral, linear) to a more favourable support configuration [6–8]. The retention and the stability of PRDPs are better if both quadrants of the jaw show abutments on strategically important areas such as the canine and posterior teeth [9]. This may protect the remaining teeth from overload and reduce possible rotational movements of the RPDP [9]. When an incisor is the terminal abutment in the quadrant, distal implants can reduce the use of retentive elements such as clasps, which provides better aesthetics and periodontal stability [8–10].

For the combination of teeth and implants to support RPDPs, either double crowns [7, 8, 11, 12] or resilient ball-attachments [6, 10, 13–19] were used on standard-diameter implants (>3.5 mm).

Standard-diameter implants require a sufficient width of the alveolar ridge (>5.5 mm). Otherwise, bone augmentation procedures are indicated, which would increase the risk of possible side effects and increase costs and treatment duration [20].

Mini-implants (diameter < 3.0 mm) are suggested to be a prosthodontic alternative to standard-diameter implants for solutions in patients with narrow alveolar ridges [20–25]. Additional advantages of mini-implants are the simplified treatment procedures with a flat learning curve, low cost, and the possible flapless surgical procedure which can decrease the post-surgical morbidity [20]. With one exception [26], mini-implants used for the retention of removable prostheses are usually one piece with a retentive ball-attachment. Therefore, no-load osseointegration is not achievable. The female matrices (housings with plastic O-rings) can be immediately polymerised into the prostheses after placement of implants with sufficient primary stability. Mini-implants are mainly used for the stabilization of complete dentures. However, long-term survival data for mini-implants are lacking and acceptable short-term survival rates (>90 %) of mini-implants are only documented for mandibular overdentures [20, 27, 28]. The short-term survival rates of immediate loaded mini-implants that are used to stabilize maxillary overdentures were unacceptably low and ranged between 54 % and 85 % [21–23]. The mean radiographic bone loss was >5 mm in the first year and therefore higher than in studies on mandibular overdentures with mean bone loss rates between 0.4 and 1.2 mm [24, 28]. In a multi-center study [25], the 4-year survival rate of mini-implants for complete denture stabilization was about 95 % without significant differences between the maxilla and mandible. In that study, all MDIs in the receiving jaw were immediately restored by rebasing the dentures with a soft liner when the insertion torque of one MDI was < 35 Ncm. The housings were picked-up after 3–4 months. The mean bone loss was insignificantly higher in the maxilla (0.8 mm) than in the mandible (0.5 mm) [29]. In another study on mini-implant supported mandibular overdentures, delayed loading appeared to be preferable to immediate loading regarding implant survival and bone loss [30]. Sound data of mini-implants as strategic abutments for a better retention of PRDPs are not available [20, 23, 27].

## Methods/design

### Study aim and design

The objectives of this study are to show that (1) immediately loaded (with housings)/restored (soft relining) mini-implants show more bone loss and less success than delayed loaded strategic mini-implants, (2) strategic mini-implants improve patients’ quality of life, patients’ satisfaction, chewing function, retention of the denture, and periodontal health of remaining abutment teeth. However, the improvements in the immediately loaded group will occur faster than in the delayed loaded group.

The protocol of this multi-center randomized parallel-group clinical trial follows the SPIRIT guidelines [31]. The study was designed according to the Good Clinical Practice guidelines (ICG-GCP) and the principles of the Declaration of Helsinki as revised in 2008.

### Study setting and participants

The Dental School at the Greifswald university hospital and three German private dental practices specializing in dental implantology and prosthodontics participate.

Study participants had to meet the following inclusion criteria:

- At least 2-months-old double crown-retained or clasp-retained PRDP in the maxilla and/or mandible that shows an unfavourable number and distribution of remaining abutment teeth in at least one quadrant of the study jaw (see classification Table 1).

**Table 1:**
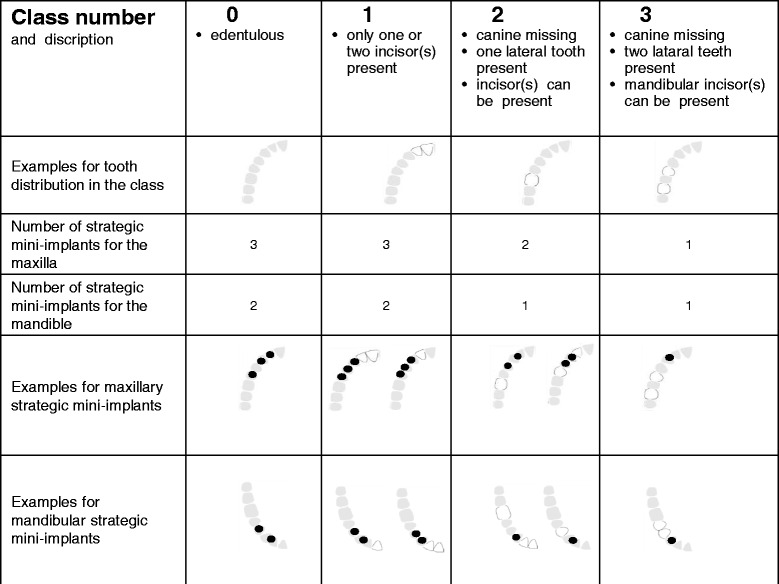
Classification for strategic mini dental implants on quadrant level
- The abutment teeth need to have healthy periodontal conditions (pocket dephts ≤ 4 mm, no bleeding on probing, attachment loss < two third of the root length, mobility grade ≤ 2).
- Vital or endodontically treated abutment teeth with a sealed root filling to the apical region without apical periodontitis.
- No contraindication for implantation, and sufficient bone in the study jaw to place an implant without augmentation procedures
- Written informed consent to participate in the study.

Exclusion criteria for participation were

- Contraindication for implantation without augmentation caused by local bone deficits
- Patients who are satisfied with their PRDP
- Patients who refuse randomizations to one of the study groups
- Poor general health, e.g. Class III-IV according to the classification of the American Society of Anaesthesiology (ASA), severe renal/or liver disease, history of a radiotherapy in the head region, chemotherapy at the time of surgical procedure, non-compensated diabetes mellitus, HIV,
- Ongoing intravenous bisphosphonate therapy
- Mental disorders (anamnestic)
- Drug abuse (anamnestic)
- Active periodontal disease and/or poor oral hygiene (mean plaque index and/or mean sulcus bleeding index ≥ 1)

### Interventions

Dentists of the centers with experience in dental implantology for more than ten years, who are familiar with the mini-implant system, perform the surgical and prosthetic treatment. Standard operating procedures were specified in a manual and imparted during the first calibration meeting. The first implant placement in each center was supervised by the treatment coordinator (TC) of the leading center.

**Fig. 1:**
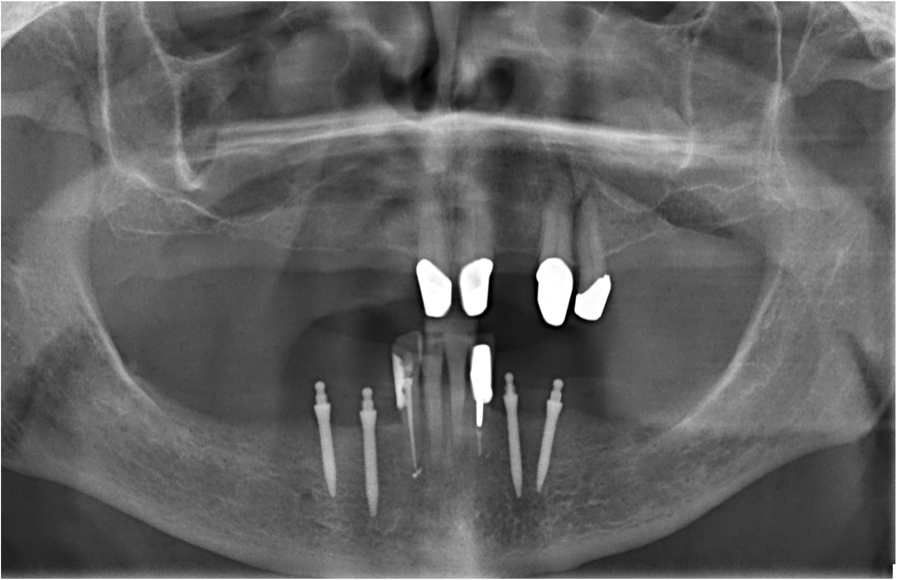
Pantomographic image of 4 mandibular strategic mini dental implants

**Fig. 2:**
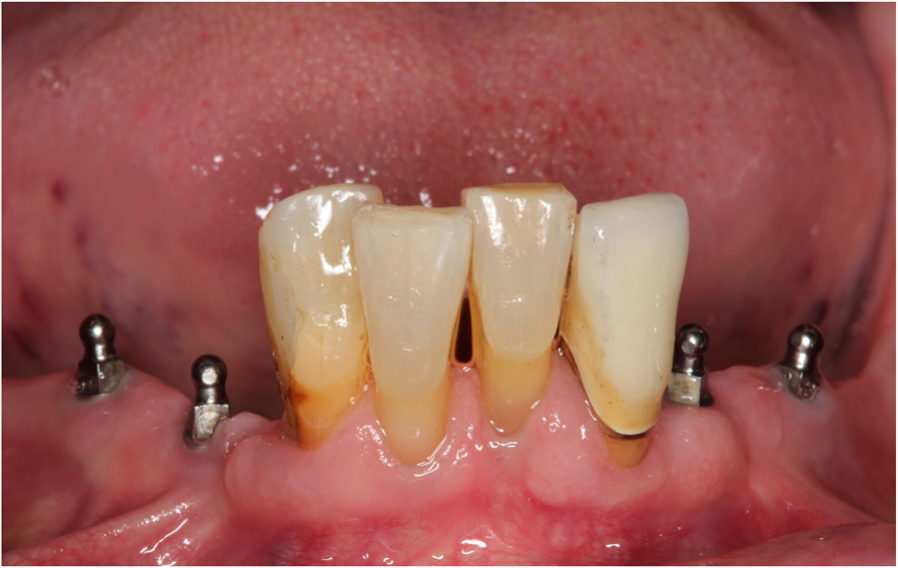
Mini dental implants presented in Fig. 1 after two weeks

Mini dental implants (MDI, 3 M ESPE, Seefeld, Germany) with lengths of 10 to 18 mm and diameters of 1.8 mm, 2.1, and 2.4 mm are used for the stabilization of PRDPs (Figs. 1 and 2). The total number of abutments per quadrant (with unfavourable distribution and number of remaining abutment teeth) will be increased to at least 3 in the maxilla or at least 2 in the mandible by the insertion of strategic MDIs (Table 1). The posterior MDI should be placed at the most posterior area of the dental arch according to the local bone volume. In the mandible, the MDIs should be located always mesial of the mental foramen. Whenever possible, the region of the canine should be occupied by an MDI (alternatively the regions of the first premolar and the lateral incisor should show abutments after intervention). If the insertion torque of one implant is < 15 Ncm due to poor bone quality the patient must be excluded from the study. Recesses for female matrices (housings) were prepared in the existing PRDP. Thereafter, a sealed envelope with the randomization detail was opened and the patient was allocated either to test group A (immediate loading/restoration) or control group B (delayed loading).

**Fig. 3:**
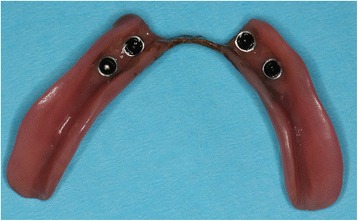
Housings for the implants presented in Fig. 2 were picked up after 4 months

Test group A (immediate loading/restoration):

If the insertion torque of all MDIs in the study jaw was ≥ 35 Ncm implants were immediately loaded. The female housings were seated on the ball attachments and picked-up using self-cured acrylic resin (Secure hard pick-up kit, 3 M ESPE, Seefeld, Germany). If the insertion torque of one MDI in the study jaw is < 35 Ncm all MDIs of this jaw were immediately restored by relining the dentures with a soft material (Secure soft reline kit, 3 M ESPE, Seefeld, Germany) embracing the ball heads. After 4 months, the soft base were substituted with the housings and acrylic resin (Fig. 3).

Control group B (delayed loading):

The recesses for the female housings in the PRDP remained empty for 4 months. Thereafter, the housings were seated on the ball attachments and picked-up using self-cured acrylic resin (Secure hard pick-up kit, 3 M ESPE, Seefeld, Germany). In the case of mucosal hyperplasia around the implant before to the fourth months, the recesses should be relined earlier using the soft material.

During the study period and thereafter the participants will be treated by the dentists of the study sites according to their dental healthcare needs. Treatments could arise from study-related events, e.g. implant or tooth loss, prosthesis fractures or study-independent events, e. g. in the opposite jaw. Treatments before study closure will be considered as outcomes.

### Outcomes

**Fig. 4:**
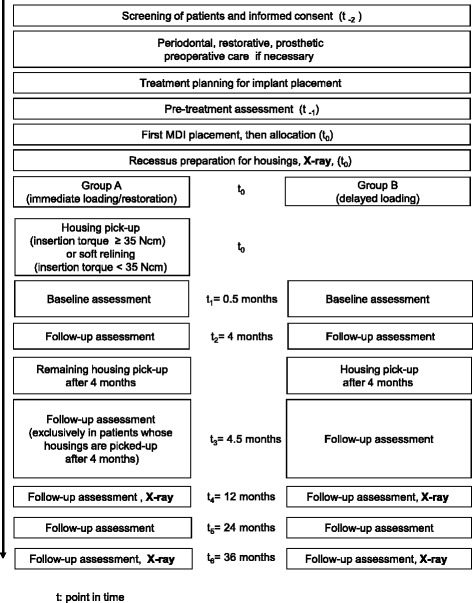
Organization chart of the trial

Crestal bone levels as the primary outcome of this trial will be determined at the 1- and 3-year follow-ups by panoramic radiographs and compared with the level immediately after implant placement (Fig. 4). Implant success is the secondary outcome according to the modified criteria by Albrektsson et al. [32]: (a) implant in situ; (b) clinical immobility of the implant; (c) no evidence of peri-implant radiolucency; (d) bone loss less than 1.5 mm for the first year and less than 0.2 mm annually after the first year of service; (e) no persistent pain, discomfort or infection. The hypothesis is tested whether immediate loaded MDIs of the test group show more bone loss and less success than the MDIs of the control group.

Tertiary outcomes are

1. Periodontal and peri-implant conditions (probing depths, bleeding on probing (BOP), modified plaque index, modified sulcus bleeding index (mod. SBI), tooth and implant mobility measured by Periotest and Osstell)
2. Oral health related quality of life (measure: short German version of the oral health impact profile (OHIP-G14) [33] and Patient’s satisfaction with the PRDP (Likert scale: totally satisfied - not at all satisfied) [34]
3. Food frequency and avoidance questionnaire will be completed by the patients to evaluate the nutrition of the patients [35]
4. Chewing efficiency will be quantified with colour-mixing ability test using a two coloured chewing gum (optoelectronical.evaluation of the ratio of unmixed colour pixels to the total pixel number in a fixed size template) [36]
5. Dental and prosthetic complications: tooth loss, caries, endodontic treatment, implant- or tooth fracture, prosthesis fracture, loss of retention, housing detachments, loss of O-rings.

We hypothesized that after the placement of MDIs there will be significant improvements only in group A and not in group B at the baseline and at the 4- month follow-up examination. After the pick-up of the housings in group B there will be also significant improvements for patients of this group.

Examinations for all secondary outcomes are scheduled before implant placement (pre-treatment, exclusively outcomes No.1- 4), 14 days after implant placement (baseline) and 4, 12, 24 and 36 months after implant placement (Fig. 4). In addition, patients whose housings are picked-up after 4 months will be examined 14 days after the pick-up. A trained examiner of the leading treatment center not involved in the treatment of participants will perform all examinations. Additionally, a second examiner will survey the bone levels at implants on the radiographs.

### Stopping criteria

A participant will be excluded from the trial if (a) the insertion torque of one implant is <15 Ncm; (b) the patient shows intolerance/allergy to titanium; (c) the occurrence of any serious adverse events related to the implantation; (d) degradation in the health status that compromises the participation in the trial; (e) failure to comply with trial requirements; (f) the patient withdraws her/his consent. The examiner and the dentists of the treatment centers are committed to report the principal investigator on the occurrence of events promptly.

The study will be terminated if (a) more than 20 % of the implants fail in any group after implant placement; (b) recruitment will not reach 80 % of the estimated number of patients during the first year; (c) the response after the first year is < 80 % of included participants.

The criteria will be checked every six months after the inclusion of the first patient. Premature termination of the trial should be decided by principal investigator in agreement with the sponsor.

### Sample size

The power calculation is based on bone loss data in a randomized study on mandibular overdentures [37] similar to the present study. Delayed loaded regular-diameter implants of 15 patients showed bone loss of 0.51 ± 0.39 mm after one year and of 0,62 ± 0.44 mm after 3 years. Immediate loaded implants of 15 patients showed bone loss of 0.91 ± 0.63 mm after 1 year and 0.98 ± 0.64 mm after 3 years. A sample size of 26 in each group will have 80 % power to detect a difference in means of 0.4 (the difference between a group 1 mean of 0.9 and a group 2 mean of 0.5) assuming that the common standard deviation is 0.5 using a two group t-test with a 0.05 two-sided significance level. Considering response rate of 75 %, a total of 80 patients (40 per group) should be allocated to the trial.

### Randomization and blinding

The randomization was performed centrally by the trial statistician based on a sequence of computer-generated numbers with a 1:1 allocation ratio stratified by jaw (maxilla, mandible) and study center.

Due to the apparent treatment differences, it is not possible to blind the dentists, clinical investigator, and/or the participating patient. However, the two examiners of the radiographs and the statistician will be blinded.

### Data management

The data of the paper case report forms will be entered in the electronical spreadsheet by assistants of the leading study site. Non-numeric data will be coded not only for statistical analyses but also to identify missing or erroneous values by range checks. The cases are pseudonymized (continuously counted according to a single main list). Each participating center got an identification number (ID-CENTER). Subjects included in the cohort got a pseudonym (ID-PROBAND) by the participating center. The centers are the keyholders for the patients’ personal data. The list of pseudonyms and patients´ ID will be listed in a special file. Only the head of the department in the dental school (keyholder) could open the pseudonyms. The principal investigator will have access to the cleaned dataset. Other members of the research group will be given access to data for analyses and publication by request.

Participant forms will be stored in locked office cabinets. The case report forms will be archived for 15 years after study closure. A member of our board of the community medicine research net with expertise in statistical methodology will guarantee the abidance of randomization and quality assurance at the data acquisition and data base. Every 12 months the member and the sponsor will forward interim reports without any analyses.

### Statistical methods

Because of the statistical power, we choose the continuous variable bone level to be the primary outcome measure. We differentiate confirmatory and exploratory analyses. Confirmatory analysis is related to differences between groups whereas exploratory analysis is related to the rate of change between groups (interaction between time and group), which yields low power test herein. However, we present the joint test of the factor group and the interaction between time and group. The explanations of the variables are outlined in Table 2.

**Table 2:** Variables, measures, hypotheses, and methods of analysis

Variable	Measure, parameterization	Variable name for syntax	Level	Hypothesis	Points in time	Methods of analysis: Stata command (version 14)
1. Primary outcome						
a) Bone level	Radiographic bone levels around implants (mesial, distal, mm, continuous)	Bone level	Implant	Group A will show more bone loss than group B	(t_0_), t_4_, t_6_	Mixed model for continuous responses: mixed
2. Secondary outcome: Implant success	Modified criteria of Albrektsson (binary): composite variable on implant level	Success	Implant	Group A will show less success than group B	t_4_, t_6_	Mixed model for binary responses: melogit
a) Related to infection	Modified sulcus bleeding index (0–3 on 2 sites per implant)	SBI	Implant		t_1_, t_2_, t_3_, t_4_, t_5_, t_6_	Mixed model for ordinal responses: meologit
	Bleeding on probing (binary on 4 sites per implant)	BOP	Implant		t_1_, t_2_, t_3_, t_4_, t_5_, t_6_	Mixed model for binary responses: melogit
b) Clinical immobility	Clinical immobility of the implant (binary)	Mobility	Implant		t_1_, t_2_, t_3_, t_4_, t_5_, t_6_	Mixed model for binary responses: melogit
c) Pain	Persistent pain or discomfort (binary)	Pain	Implant		t_1_, t_2_, t_3_, t_4_, t_5_, t_6_	Mixed model for binary responses: melogit
d) Radiolucency	Evidence of peri-implant radiolucency (binary)	XLucency	Implant		t_4_, t_6_	Mixed model for binary responses: melogit
e) Survival	Implant in situ (binary)	ImplantLoss	Implant		Continuous time	Kaplan-Meier
3. Tertiary outcome						
a) Periodontal and periimplant conditions	Probing depths (mm, continuous on 4 sites per implant)	ProbingDepth	Implant	Group A will show higher values than group B	t_1_, t_2_, t_3_, t_4_, t_5_, t_6_	Mixed model for continuous responses: mixed
	Probing depths (mm, continuous on 4 sites per tooth)	ProbingDepth	Tooth	Overall improvement at teeth, improvement occurred faster in group A than in group B	t_1_, t_2_, t_3_, t_4_, t_5_, t_6_	Mixed model for continuous responses: mixed
	Osstell (0–100, continuous on implant level)	Osstell	Implant	Group A will show lower values than group B until the fourth month, thereafter equalization between A and B	t_1_, t_2_, t_3_, t_4_, t_5_, t_6_	Mixed model for continuous responses: mixed
	Periotest (−8.0 - +50.0, continuous on tooth level)	Periotest	Tooth	Overall improvement at teeth, improvement occurred faster in group A than in group B	t_1_, t_2_, t_3_, t_4_, t_5_, t_6_	Mixed model for continuous responses: mixed
	Periotest (−8.0 - +50.0, continuous on implant level)	Periotest	Implant	Group A will show higher values than group B until the fourth month, thereafter equalization between A and B	t_1_, t_2_, t_3_, t_4_, t_5_, t_6_	Mixed model for continuous responses: mixed
b) Oral health related quality of life	OHIP-G14 questionnnaire (0–56, continuous)	OHIP	Patient	Overall improvement, improvement occurred faster in group A than in group B	t_1_, t_2_, t_3_, t_4_, t_5_, t_6_	Mixed model for ordinal responses: meologit
c) Patient’s satisfaction with the PRDP	Questionnnaire 8 items (Five-point Likert-scale, 8–40 continuous)	Satisfaction	Patient	Overall improvement, improvement occurred faster in group A than in group B	t_1_, t_2_, t_3_, t_4_, t_5_, t_6_	Mixed model for ordinal responses: meologit
d) Nutrition of the patients	Food frequency questionnaire (1–7)	FFQ	Patient	Overall improvement, improvement occurred faster in group A than in group B	t_1_, t_2_, t_3_, t_4_, t_5_, t_6_	Mixed model for ordinal responses: meologit
	Food avoidance questionnaire (binary)	FAQ	Patient	Overall improvement, improvement occurred faster in group A than in group B	t_1_, t_2_, t_3_, t_4_, t_5_, t_6_	Mixed model for binary responses: melogit
e) Chewing efficiency	Colour-mixing ability test with two coloured chewing gum (continuous)	Chewing	Patient	Overall improvement, improvement occurred faster in group A than in group B	t_1_, t_2_, t_3_, t_4_, t_5_, t_6_	Mixed model for ordinal responses: meologit
4. Exposure						
Group	2 categories		Jaw		t_0_	
5. Time variables						
TimePoint	0-6 for outcomes				0-6 for t_0_ –t_6_	
Week	Time [weeks]		Patient		Week	
SqrtWeek	Square root of week		Patient		Root of week	
Time			Patient		Continuous	
6. Confounder						
Age	Restricted cubic splines with 3 knots (2 coefficients)	Age	Patient		t_−1_	
Gender	2 categories (men; women)	Gender	Patient		t_−1_	
Center	4 categories	Center	Patient		t_−1_	
Jaw class	4 categories	JawClass	Jaw		t_−1_	
Jaw	2 categories (upper; lower)	Jaw	Jaw		t_−1_	
Tooth	1-16 within jaw	Tooth	Tooth		t_−1_	
Site	Up to 4 sites	Site	Site			
Smoking	3 categories (never; ex; current)	Smoking	Patient			
School education	3 categories (<10, 10, >10 years)	Education	Patient		t_−1_	
Probing depth	Restricted cubic splines with 3 knots (2 coefficients)	ProbingDepth0	Tooth		t_−1_	
Bone level	Before treatment in groups	BoneLevel0			t_0_	
Periotest	Restricted cubic splines with 3 knots (2 coefficients)	Periotest0	Tooth		t_−1_	
OHIP-G14 questionnnaire (0–56, continuous)	Restricted cubic splines with 3 knots (2 coefficients)	OHIP0	Patient		t_−1_	
Questionnnaire 8 items (Five-point Likert-scale, 8–40 continuous)	Linear term only	Satisfaction0	Patient		t_−1_	
Food frequency questionnaire (1–7)	Linear term only	FFQ0	Patient		t_−1_	
Food avoidance questionnaire (binary)		FAQ0	Patient		t_−1_	
colour-mixing ability test with two coloured chewing gum (continuous)	Linear term only	Chewing0	Patient		t_−1_	
7. Subgroup analysis						
Jaw class 0 vs 1-3	Secondary outcomes	JawClass		Improvement in group A is better than in group B	t_−1_	
8. Additional analysis						
Maxilla vs. mandible	All outcomes			Maxilla will show less success and more bone loss than mandible; Implant stability (Periotest, Osstell) is lower in the maxilla than in the mandible; no differences in the improvement of other secondary outcomes	t_−1_	

t_1_: 0.5 months; t_2_: 4 months; t_3_: 4.5 months; t_4_: 12 months; t_5_: 24 months; t_6_: 36 months

### Primary outcome: bone level

Because jaw was used for randomization, we extend Gilthorpe’s mixed model by inserting jaw to levels for patient, tooth, and site [38] and adjust for model complexity by using the Kenward-Roger correction [39]. To ensure hierarchical levels, tooth positions within each jaw are coded from 1 to 16 instead of from 1 to 32 on patient level. The first adjustment set consists of risk factors of periodontal disease including age, gender, and smoking [40]; and design variables including centre, jaw [41], jaw classification, and time. Because X-ray at t_0_ is assessed before differentiating treatment in groups, it is a baseline measurement which can additionally be used to increase efficiency [42]. Note also the discussion on pages 158–160 in Harrell’s textbook [43]. The syntax of the basic confirmatory model with the highest statistical power for the factor group is (Stata’s version 14):



Here, only the time points t_4_ and t_6_ are used as dependent variable (Table 2). To interpret changes after 12 and 36 months in exploratory analyses, we include t_0_ as dependent variable:



In sensitivity analyses, we allow for continuous time instead of points in time, for correlated random effects, or for random effects of other than tooth level and suspend the Kenward-Roger correction. In the second adjustment set, jaw classification is replaced by patient’s mean probing depth before randomization; in the third and fourth set, school education will be added to the first and second set, respectively.

### Secondary outcomes

To avoid false positive conclusions, we state a priority ordering in advance [44] based on information on scale (continuous/ordinal/binary), number of time points, level (site/tooth/jaw/patient), and expected number of events (see Table 2).

For six time points, we allow for nonlinear change. The nonlinear change is modelled by taking the square root of time [45] because we expect a higher rate of change in the first weeks. The confirmatory and the exploratory SBI model:





### Tertiary outcomes

See Table 2 for priority ordering. We allow for nonlinear change as described above. If possible we adjust for values before treatment [40, 43]. The confirmatory and the exploratory model of periotest for teeth:





For tertiary outcomes other than periodontal or peri-implant conditions, we expect differences between groups before housing in group B (time points t_1_ and t_2_) but no differences after that (time points t_4_-t_6_). Thus, we present only confirmatory analyses. The model for food frequency questionnaire at time points t_1_ and t_2_:



### Per protocol set

We define the per-protocol by the following criteria: only one study jaw per patient; minimum number of implants as indicated in Table 1; no poor general health, no bisphosphonate therapy, no mental disorder, and no drug abuse during follow-ups; X-ray examination after 36 months is available.

### Evaluation of safety and tolerability

Because the sample size on patient level is low, we do not analyse differences between groups but describe vital signs and clinical adverse events with type, time, and severity.

### Additional notes

We do not impute missing data for two reasons. First, we choose mixed models, which are robust to missing data at random [45]; second, an imputation on four levels for this small sample of about 80 patients can hardly be justified [46]. For model checking and sensitivity analysis we use a variety of methods [45, 47]. For subgroup analysis of jaw classification, the two-sided alpha level will be reduced from 0.05 to 0.025.

An interim analysis will not be done. A statistician (CS) blinded to the study groups will perform the calculations by using up-to-date versions of STATA (StataCorp LP, College Station, Texas, USA) and R for windows, Version 2.12.2 (R Foundation for Statistical Computing, Vienna, Austria).

### Recruitment

Suitable patients were recruited via personal information during standard recall in two steps. Patients with a suitable dental status were asked whether they were satisfied with their PRDP. After signed informed consent, patients that are unsatisfied with their RPDP were screened according to the inclusion and exclusion criteria. Patient with treatment need, i.e. technically inacceptable RPDP, periodontitis, caries, but who otherwise met the inclusion criteria received firstly an adequate dental and/or prosthodontic treatment, Thereafter, they were re-examined.

All patients were evaluated by the calibrated examiner. Patients meeting the dental, medical, and prosthodontic inclusion criteria, were radiographically examined using panoramic X-ray with a reference marker (titanium tube, steel pin, steel ball) to determine whether the residual bone of the jaw meets the inclusion criterion: to place the MDIs without augmentation procedures. In order to achieve comparable treatment plans, the TC assessed the X-rays and pseudonymized documents of each patient to check and, if necessary, to correct the original treatment plan of all centres. If all inclusion criteria were met, patients were included in the study. In addition to the pre-treatment examination, the baseline characteristics (age, gender, medical history, smoking habits, oral health behaviour, school education, household income) of the study participants were assessed by a questionnaire. The schedule of recruitment was 17 months (First Patient in 01.11.2013, last Patient in: 31.03.2015).

### Audits

Audits will be conducted to ensure data validity and reliability. The sponsor may assign independent persons (auditors) otherwise not involved in trial conduct to perform audits at the study centers. The auditors are granted permission to access study-related documents (i.e. protocol, participant files, study-related correspondence). Auditors are supposed to comply with data protection.

### Publication

Analyses, presentations and publications will include the data of all study sites and not from one center alone. Results of prespecified outcome parameters will be published and released to the general medical community and patients regardless of the magnitude or effect direction of interventions.

The lead author, the principal investigator, the examiner, the statistician and at least one author per center will be listed as authors in presentations and publications. Additional professionals can be listed in the acknowledgement section. Professional medical writers will be not deployed.

## Discussion

This randomized multi-center trial was initiated by the researchers to compare the immediate loading/restoration with delayed loading of the implants and to evaluate the suitability of MDIs as supplementary abutment for the stabilization of PRDPs regardless of the loading time.

Some limitations of this study merit consideration. Although all treating dentists of the four centers have been using dental implants for more than 10 years and are familiar with the MDI system, entrenched approaches could result in different outcomes despite of the treatment manual and calibration session. Therefore, the first interventions were supervised by the treatment coordinator of the leading center to avoid treatment differences effectively. Nevertheless, multivariate analyses will be adjusted by the study centers. The study jaws showed different distributions of remaining teeth and different types of prostheses. Therefore, the classification was developed and subgroup analyses are planned to consider the various starting positions. Because patients who are satisfied with their PRDP were excluded, this criterion might bias patients to positive answers following MDI placement. However, the desire for dental implants is very weak among satisfied patients. Therefore, overtreatment should be avoided.

Nevertheless, this is the first study to evaluate the effectiveness of MDI for the stabilization of RPDP. The therapy approaches are standardized as far as possible. The examinations are performed by a dentist who is not involved in the treatment of the patients.

The idea of this clinical trial was to compare two different loading protocols and not two different treatment alternatives, e.g., the placement of standard-diameter implants versus mini-implants or no implant placement versus mini-implants. No implant placement would imply ethical conflicts and response problems after randomization. Standard-diameter implants require sufficient bone volume. Therefore, in narrow alveolar ridges, augmentative procedures would be necessary.

Before recruitment of study participants, the minimum number must be estimated to detect a statistically significant difference between the groups as in the present study. The sample size calculation requires assumptions that are typically cannot really be tested until the data have been collected. Sample size calculations are thus inherently hypothetical [48]. The number of additional subjects to consider dropouts over the study period is based on the experience of researchers [49]. The sample size for this trial required a multi-center design. The estimated loss to follow-up rate of 25 % is rather conservative and should also consider stopping criteria such as a low insertion torque of the MDI (<15 Ncm).

The study aims for a number of secondary endpoints including biological and technical complications, chewing efficiency and patient-based outcomes such as satisfaction and quality of life.

The chewing efficiency is measured by a mixing ability test of two-coloured chewing gum, to test the hypothesis that the additional mini-implant support of a tooth-retained PRDP improves the degree of mixing of the two colours after 20 chewing strokes [36]. For the quantitative evaluation of the flattened and scanned chewing gum specimens, a software was developed for a fast, simple and valid extraction of clinically oriented conclusions [50].

The oral health impact profile (OHIP) is a valid and reliable instrument for the measurement of the oral health related quality of life (OHRQoL) [15, 33]. A significant improvement in the OHRQoL was reported after the placement of standard-diameter implants under existing PRDPs in only one prospective study [15]. Nonetheless, there are some studies that showed some significant increase in patient satisfaction after PRDP stabilization using standard implants [10, 13, 14, 17]. In another study, distal strategic implants under PRDPs significantly improved the masseter muscle thickness and the maximum bite force [18]. The same research group further reported a particle size reduction after the chewing of silicone test cubes, and a better nutrient intake following the insertion of strategic implants [19]. Many of other studies on strategic standard implants for PRDP stabilization evaluated clinical outcomes exclusively [6–8, 11, 12, 51–53]. The 3- to 8-year-survival rates ranged between 90 and 100 % for implants and between 93 and 100 % for PRDPs.

As mentioned previously, a number of studies showed the middle- and long-term behaviour and the positive effect of strategic implants on the performance of PRDPs and patient’ satisfaction in recent years. However, some limitations of standard-diameter implants such as high costs, placement effort and treatment duration could be compensated by using mini-implants. Therefore, prospective studies are needed to evaluate the clinical performance and the treatment effect of mini-implants in this indication.

### Trial status

At the time of submission of this paper, the patient recruitment and the implant placement was finished.

## Abbreviations

BOP, bleeding on probing; ID, identification; MDI, mini dental implant; mod. SBI, modified sulcus bleeding index; OHIP-G14, oral health impact profile, Germany, 14 items; OHRQoL, oral health related quality of life; PRDP, partial removable dental prostheses; SPIRIT, standard protocol items, recommendations for interventional trials; TC, treatment coordinator

## Additional file

Additional file 1: Model consent form given to the participants (In German). (PDF 342 kb)
