# Supplementary material for: Immediate versus delayed loading of strategic mini dental implants for the stabilization of partial removable dental prostheses: a patient cluster randomized, parallel-group 3-year trial
Source: BMC Oral Health. 2016 Jul 30;17:30. doi: 10.1186/s12903-016-0259-z (PMC4967347; doi:10.1186/s12903-016-0259-z)
Supplement: Additional file 1: — Model consent form given to the participants (In German). (PDF 342 kb) [file 12903_2016_259_MOESM1_ESM.pdf]

**Prüfeinrichtung:**	Universitätsmedizin Greifswald<br>Poliklinik für Zahnärztliche Prothetik, Alterszahnmedizin und medizinische Werkstoffkunde<br>Rorgerberstr. 8<br>17475 Greifswald<br>Tel.: 03834 867140
**Prüfstelle:**	Universitätsmedizin/Zahnarztpraxis
**Studienleiter:**	OA PD Dr. Torsten Mundt
**Koordinator Studien:**	OA Dr. Thomas Klinke
**Prüfarzt:**	ZA Ahmad Al-Jaghsi, DDS, MSc

Strategische Mini-Dental-Implantate (MDI) zur Stabilisierung von herausnehmbaren Teilprothesen.

Sehr geehrte Patientin, sehr geehrter Patient,

wir möchten Sie fragen, ob Sie bereit sind, an der nachfolgend beschriebenen klinischen Studie teilzunehmen.

Um wissenschaftlich fundierte Aussagen darüber treffen zu können, wie erfolgreich ein Implantatsystem über einen längeren Zeitraum ist, ist es wichtig, dass in bestimmten Zeitabständen die Implantate und die damit verankerte Prothesen untersucht werden. Die klinische Studie, die wir Ihnen hier vorstellen, wurde – wie es das Gesetz verlangt – von einer Ethikkommission zustimmend bewertet und bei der zuständigen Behörde angezeigt. Die Studie wird veranlasst und organisiert von der Poliklinik für Zahnärztliche Prothetik, Alterszahnheilkunde und medizinische Werkstoffkunde der Universität Greifswald und teilfinanziert durch den Implantathersteller (3M ESPE Dental Products, Seefeld).

Ihre Teilnahme an dieser Untersuchung ist freiwillig. Sie werden in diese Studie also nur dann einbezogen, wenn Sie dazu schriftlich Ihre Einwilligung erklären. Sofern Sie nicht an der klinischen Studie teilnehmen oder später aus ihr ausscheiden möchten, erwachsen Ihnen daraus keine Nachteile.

Sie wurden bereits auf die geplante Studie angesprochen. Der nachfolgende Text soll Ihnen die Ziele und den Ablauf erläutern. Anschließend wird ein Prüfarzt das Aufklärungsgespräch mit Ihnen führen. Bitte zögern Sie nicht, alle Punkte anzusprechen, die Ihnen unklar sind. Sie werden danach ausreichend Bedenkzeit erhalten, um über Ihre Teilnahme zu entscheiden.

1. Warum wird diese Studie durchgeführt?

Wenn an einem stark reduzierten Restzahnbestand eine Prothese verankert wird, reicht oftmals die Anzahl und Position der Zähne nicht aus, der Prothese einen zufriedenstellenden Halt und Lagestabilität zu bieten. Zusätzliche Implantate, die mit ihren Halteelementen nachträglich in die vorhandene Prothese eingearbeitet werden, stabilisieren die Prothese nicht nur sondern entlasten auch den Restzahnbestand
Für diese Indikation scheinen die einteiligen durchmesserreduzierten Implantate, die wir bei Ihnen einsetzen möchten (Mini Dental Implants, MDI, 3M ESPE, Seefeld, Deutschland) besonders gut geeignet, da sie auch bei schmalen Kieferkamm gewebeschonend inseriert werden können. Da die Prothese erhalten bleibt, ist dieses Vorgehen besonders kostengünstig und Sie müssen sich nicht an einen neuen Zahnersatz gewöhnen.
Untersuchungen von Implantaten und implantatgetragenen Prothesen helfen, Faktoren, die sich im Laufe der Tragedauer negativ auf die Implantate oder die Prothese auswirken könnten zu identifizieren. Darüber hinaus können Rückschlüsse für eine noch optimalere Implantatversorgung gezogen werden. Für die Analyse dieser Einflussfaktoren benötigen wir auch allgemeinmedizinische Angaben aus Ihrer Krankengeschichte (Anamnese) und über Ihre Lebensgewohnheiten z. B. das Rauchen. Weiterhin soll mit unserer Studie ermittelt werden, ob es Unterschiede gibt zwischen einer Sofortbelastung und einer Spätbelastung der Implantate. Deshalb werden die Implantate von der Hälfte der Studienpatienten sofort mit der Prothese verbunden (entweder mit einer weichbleibenden Unterfütterung oder die druckknopfartigen Haltelemente werden sofort in die Prothese eingearbeitet), bei der anderen Hälfte der Patienten verbleiben die Implantate für ca. 4 Monate weitestgehend unbelastet unter der Prothese und die Halteelemente werden erst danach eingearbeitet. Beide Therapievarianten sind inzwischen wissenschaftlich anerkannt, wurden jedoch in dieser Indikation bisher noch nicht direkt miteinander verglichen.

2. Wie ist der Ablauf der Studie?

Falls Ihre Lückengebisssituation zutrifft und Sie mit dem Halt Ihrer Prothese unzufrieden sind, wird untersucht an welchen Positionen Implantate möglich sind. Nach einer allgemeinen Anamnese, die mögliche Risikofaktoren für Zahnimplantate ermitteln soll, werden Sie zunächst genauer klinisch und röntgenologisch untersucht, ob Sie für die Studie in Frage kommen.

Stehen die Implantatpositionen fest, erhalten Sie einen Heil- und Kostenplan. Vor der Implantation erfolgen eine genaue Untersuchung der vorhandenen Zähne und die exakte Ausmessung des Knochenangebotes. Sie werden zu Rauch-, Mundhygiene-und Ernährungsgewohnheiten befragt. Außerdem wird mit einem Kaugummi Ihr Kauvermögen vor der Implantation getestet. Weitere Fragebögen zielen auf die Zufriedenheit mit Ihrer Mundsituation und mit Ihrer Prothese. In der folgenden Sitzung werden die Implantate unter lokaler Anästhesie inseriert, der Kiefer geröntgt und die Prothese für die Aufnahme der Halteelemente vorbereitet. Danach wird ausgelost, ob die Implantate sofort (Therapiearm A) oder nach vier Monaten (Therapiearm B) mit der Prothese belastet werden. Im Therapiearm A erfolgt die Sofortversorgung je nach Knochenqualität entweder mit einer weichbleibenden Unterfütterung (bei weichem Knochen) oder mit der unmittelbaren Einarbeitung der druckknopfartigen Haltelemente (bei festem Knochen). Nach vier Monaten werden alle Implantate über die Halteelemente definitiv mit der Prothese belastet.

Nach der Implantation werden die Implantate und die Prothesen in regelmäßigen Abständen (nach 2 Wochen, 4 Monaten, 1 Jahr, 2 Jahren, 3 Jahren) untersucht, der Kautest wiederholt und Sie werden während der jeweiligen Kontrolltermine zur Zufriedenheit mit der Mundsituation und zu Ihren Ernährungsgewohnheiten befragt. Während der 1- und der 3-Jahres-Nachuntersuchung werden Implantate, Zähne und der Kieferknochen mit einer Röntgenaufnahme zusätzlich kontrolliert. Dieses Röntgenintervall entspricht dem Standardvorgehen zur röntgenologischen Kontrolle im Rahmen der zahnärztlichen Implantattherapie. Für den Erfolg der Studie ist es unerlässlich, dass Sie alle Termine wahrnehmen, andernfalls können Ihre Daten nicht verwendet werden.

Wenn Sie von anderen Zahnärzten/Ärzten behandelt werden, müssen Sie diese über Ihre Teilnahme an der klinischen Studie informieren. Auch Ihr Prüfarzt muss über jede zahnmedizinische Behandlung, die Sie durch einen anderen Zahnarzt während der klinischen Studie erhalten, informiert werden.

3. Welchen persönlichen Nutzen habe ich von der Teilnahme an der Studie?

Ihre Prothese bekommt durch die Implantate höchstwahrscheinlich einen besseren Halt und mehr Stabilität, so dass Ihr Kauvermögen optimiert wird. Die Implantate und die Verbindungselemente zur Prothese erhalten Sie zu einem Vorzugspreis, da die Implantatteile Ihnen nicht in Rechnung gestellt werden. Die regelmäßigen Nachkontrollen dienen auch dazu, mögliche Probleme mit Zähnen, Implantaten und Prothesen frühzeitig zu erkennen und zu beseitigen.

4. Welche Risiken sind mit der Teilnahme an der Studie verbunden?

Während der Implantation kann es zu Nervverletzungen (insbesondere im Unterkiefer), Schädigung von Nachbarzähnen und zu Nasenboden- und Kieferhöhlenverletzungen (im Oberkiefer) kommen. Weitere Risiken sind– wie bei jeder anderen Operation – die nachfolgend aufgeführten allgemeinen Risiken: Störungen des Nervensystems, Änderungen des Blutdrucks oder des Pulses, Blutungen, entzündlichen Prozesse, Wundinfektionen, allergische Reaktionen, Wundheilungsstörungen bis hin zum Implantatverlust, Narbenbildung und Thrombosen bzw. Embolien.

Durch die Vorbereitung der Prothese zur Aufnahme der Halteelemente wird der Prothesenkörper zunächst geschwächt, wodurch die Prothese frakturieren könnte. Die Röntgenaufnahmen zur Diagnostik und Nachkontrolle sind mit einer erhöhten Strahlenbelastung verbunden.

Bitte teilen Sie den Mitarbeitern der Prüfstelle *alle* Beschwerden, Erkrankungen oder Verletzungen mit, die im Verlauf der klinischen Studie auftreten. Falls diese schwerwiegend sind, teilen Sie den Mitarbeitern der Prüfstelle diese bitte umgehend mit, ggf. telefonisch. Unmittelbar nach lokaler Anästhesie dürfen Sie für 3-4 Std. nicht am Straßenverkehr teilnehmen oder Maschinen führen. Ferner sollten Sie für den Zeitraum der Betäubung keine Speisen zu sich nehmen, um eine Verletzung der Mundhöhle durch die Zähne zu vermeiden.

5. Wer darf an dieser klinischen Nachuntersuchung nicht teilnehmen?

Sie können an dieser klinischen Studie nur teilnehmen, wenn Sie nicht schwer erkrankt sind und sich nicht gleichzeitig für andere klinische Studien oder andere klinische Forschungsprojekte zur Verfügung stellen oder bis vor kurzem teilgenommen haben. Schwangere Frauen dürfen an der Studie nicht teilnehmen.

6. Entstehen für mich Kosten durch die Teilnahme an der klinischen Studie? Erhalte ich eine Aufwandsentschädigung?

Durch Ihre Teilnahme an dieser klinischen Studie entstehen für Sie Kosten für den operativen Eingriff und das Einarbeiten der Halteelemente.

Die Höhe der Kosten ist unterschiedlich und abhängig von der Anzahl der Implantate. Insgesamt ist der Preis für diese zahnärztliche Therapie jedoch deutlich reduziert. Sie erhalten für Ihre Teilnahme an jeder Nachuntersuchung eine kleine Aufwandsentschädigung (30 EUR).

7. Bin ich während der klinischen Studie versichert?

Bei Diagnostik und Therapie sind alle Studienteilnehmer über das behandelnde Zentrum versichert. Für die Nachuntersuchungen wurde eine Wegeunfallversicherung abgeschlossen. Der Umfang des Versicherungsschutzes ergibt sich aus den Versicherungsunterlagen, die Sie auf Wunsch ausgehändigt bekommen.
Schäden sollten Sie unverzüglich dem Versicherer:

Name und Anschrift der Versicherung:
XXX

melden, gegebenenfalls mit Unterstützung durch Ihren Prüfarzt, um Ihren Versicherungsschutz nicht zu gefährden. Sofern Ihr Prüfarzt Sie dabei unterstützt, erhalten Sie eine Kopie der Meldung. Richten Sie Ihre Anzeige direkt an den Versicherer, so informieren Sie bitte zusätzlich Ihren Prüfarzt.

Bei der Aufklärung der Ursache oder des Umfangs eines Schadens müssen Sie mitwirken und alles unternehmen, um den Schaden abzuwenden und zu mindern.
Während der Dauer der klinischen Studie dürfen Sie sich einer anderen zahnmedizinischen Behandlung – außer in Notfällen – nur nach vorheriger Rücksprache mit dem Prüfarzt

unterziehen (Vorlage Studienausweis). Von einer erfolgten Notfallbehandlung müssen Sie den Prüfarzt unverzüglich unterrichten.

8. Wer entscheidet, ob ich aus der Studie ausscheide?

Sie können jederzeit, auch ohne Angabe von Gründen, Ihre Teilnahme beenden, ohne dass Ihnen dadurch irgendwelche Nachteile bei Ihrer medizinischen Behandlung entstehen.
Unter gewissen Umständen ist es aber auch möglich, dass der Prüfarzt oder der Sponsor entscheidet, Ihre Teilnahme an der klinischen Studie vorzeitig zu beenden, ohne, dass Sie auf die Entscheidung Einfluss haben. Die Gründe hierfür können z. B. sein:

- Ihre weitere Teilnahme an der klinischen Studie ist ärztlich nicht mehr vertretbar.
- es wird die gesamte klinische Studie abgebrochen.
- Sie nehmen die geforderten Termine nicht wahr, so dass Ihre Daten nicht zu verwenden sind.

Sofern Sie sich dazu entschließen, vorzeitig aus der klinischen Studie auszuscheiden, oder Ihre Teilnahme aus einem der genannten Gründe vorzeitig beendet wird, ist es für Ihre eigene Sicherheit wichtig, dass Sie sich einer empfohlenen abschließenden Kontrolluntersuchung unterziehen.
Der Prüfarzt wird mit Ihnen besprechen, ob und wann weitere Kontrolluntersuchungen notwendig sind.

9. Was geschieht mit meinen Daten?

Während der klinischen Nachuntersuchung werden medizinische Befunde und persönliche Informationen von Ihnen erhoben und in der Prüfstelle in Ihrer persönlichen Akte niedergeschrieben oder elektronisch gespeichert. Die für die klinische Nachuntersuchung wichtigen Daten werden zusätzlich in pseudonymisierter Form gespeichert, ausgewertet und gegebenenfalls weitergegeben.

Pseudonymisiert bedeutet, dass keine Angaben von Namen oder Initialen verwendet werden, sondern nur ein Nummern- und/oder Buchstabencode, evtl. mit Angabe des Geburtsjahres.
Die Daten sind gegen unbefugten Zugriff gesichert. Eine Entschlüsselung erfolgt nur unter den vom Gesetz vorgeschriebenen Voraussetzungen.

Die gesetzlichen Bestimmungen enthalten nähere Vorgaben für den erforderlichen Umfang der Einwilligung in die Datenerhebung und -verwendung. **Einzelheiten, insbesondere zur Möglichkeit eines Widerrufs, entnehmen Sie bitte der Einwilligungserklärung, die im Anschluss an diese Patienteninformation abgedruckt ist.**

10. Was geschieht mit meinen Röntgenaufnahmen und Fotografien?

Die Röntgenaufnahme wird nach Abschluss der Nachuntersuchung Ihrer Patientenkartei entsprechend den Vorgaben der gültigen Röntgenverordnung aufbewahrt und in der Praxis zugeordnet.
Die angefertigten Fotos werden in den Prüfzentren auf unbestimmte Zeit gelagert und pseudonymisiert in der Studienzentrale aufbewahrt.

11. An wen wende ich mich bei weiteren Fragen?

Beratungsgespräche an der Prüfstelle

Sie haben stets die Gelegenheit den auf Seite 1 genannten oder einem anderen Prüfarzt zu kontaktieren, um weitere Fragen im Zusammenhang mit der klinischen Nachuntersuchung zu klären. Auch Fragen, die Ihre Rechte und Pflichten als Patient und Teilnehmer an der klinischen Nachuntersuchung betreffen, werden gerne beantwortet.

Prüfeinrichtung: Universitätsmedizin Greifswald
Poliklinik für Zahnärztliche Prothetik, Alterszahnmedizin und medizinische Werkstoffkunde
Rorgerberstr. 8
17475 Greifswald
Tel.: 03834 867140
Prüfstelle: Zahnarztpraxis
Studienleiter: OA PD Dr. Torsten Mundt
Prüfarzt: ZA Ahmad Al-Jaghsi, DDS, MSc.

Titel der Studie
Strategische Mini-Dental-Implantate (MDI) zur Stabilisierung von herausnehmbaren Teilprothesen.

Einwilligungserklärung

....
Name des Patienten in Druckbuchstaben

geb. am .... Teilnehmer-Nr. ....

Ich bin in einem persönlichen Gespräch durch den Untersucher

....
Name der Ärztin / des Arztes

ausführlich und verständlich über Wesen, Bedeutung, Risiken und Tragweite der klinischen Studie aufgeklärt worden. Ich habe darüber hinaus den Text der Patienteninformation sowie die hier nachfolgend abgedruckte Datenschutzerklärung gelesen und verstanden. Ich hatte die Gelegenheit, mit dem Untersucher über die Durchführung der klinischen Studie zu sprechen. Alle meine Fragen wurden zufrieden stellend beantwortet.

Ich hatte ausreichend Zeit, mich zu entscheiden.

Mir ist bekannt, dass ich jederzeit und ohne Angabe von Gründen meine Einwilligung zur Teilnahme an der Studie zurückziehen kann (mündlich oder schriftlich), ohne dass mir daraus Nachteile für meine medizinische Behandlung entstehen. Ein Exemplar der Patienten-Information und -Einwilligung habe ich erhalten. Ein Exemplar verbleibt im Prüfzentrum.

Datenschutz:

Mir ist bekannt, dass bei dieser klinischen Studie personenbezogene Daten, insbesondere medizinische Befunde über mich erhoben, gespeichert und ausgewertet werden sollen. Die Verwendung der Angaben über meine Gesundheit erfolgt nach gesetzlichen Bestimmungen und setzt vor der Teilnahme an der klinischen Studie folgende freiwillig abgegebene Einwilligungserklärung voraus, das heißt ohne die nachfolgende Einwilligung kann ich nicht an der klinischen Nachuntersuchung teilnehmen.

1. Ich erkläre mich damit einverstanden, dass im Rahmen dieser klinischen Studie personenbezogene Daten, insbesondere Angaben über meine Gesundheit, über mich erhoben und in Papierform sowie auf elektronischen Datenträgern in der Zahnarztpraxis ………………………… aufgezeichnet werden. Soweit erforderlich, dürfen die erhobenen Daten pseudonymisiert (verschlüsselt) an die Universitätsmedizin Greifswald zum Zwecke der wissenschaftlichen Auswertung weitergegeben werden

3. Ich bin darüber aufgeklärt worden, dass ich jederzeit die Teilnahme an der klinischen Studie beenden kann. Beim Widerruf meiner Einwilligung, an der Studie teilzunehmen, habe ich das Recht, die Löschung aller meiner bis dahin gespeicherten personenbezogenen Daten zu verlangen.

4. Ich erkläre mich damit einverstanden, dass meine Daten nach Beendigung oder Abbruch der Studie mindestens zehn Jahre aufbewahrt werden. Danach werden meine personenbezogenen Daten gelöscht, soweit nicht gesetzliche, satzungsmäßige oder vertragliche Aufbewahrungsfristen entgegenstehen.

5. Ich bin damit einverstanden, dass mein Zahnarzt .... über eventuelle krankhaften Befunde informiert wird (falls nicht gewünscht, bitte streichen).

Ich erkläre mich bereit, an der klinischen Studie freiwillig teilzunehmen.

....
Name des Patienten in Druckbuchstaben

....
Datum Unterschrift **Patient/In**

Ich habe das Aufklärungsgespräch geführt und die Einwilligung des Patienten eingeholt.

....
Name des Prüfarztes/der Prüfärztin in Druckbuchstaben

....
Datum Unterschrift des/der **Prüfarztes/in**
